# Musical activity and emotional competence – a twin study

**DOI:** 10.3389/fpsyg.2014.00774

**Published:** 2014-07-16

**Authors:** Töres P. Theorell, Anna-Karin Lennartsson, Miriam A. Mosing, Fredrik Ullén

**Affiliations:** ^1^Department of Neuroscience, Karolinska InstituteStockholm, Sweden; ^2^Stress Research Institute, Stockholm University StockholmSweden

**Keywords:** alexithymia, musicality, depression, emotional competence, twins

## Abstract

The hypothesis was tested that musical activities may contribute to the prevention of alexithymia. We tested whether musical creative achievement and musical practice are associated with lower alexithymia. 8000 Swedish twins aged 27–54 were studied. Alexithymia was assessed using the Toronto Alexithymia Scale-20. Musical achievement was rated on a 7-graded scale. Participants estimated number of hours of music practice during different ages throughout life. A total life estimation of number of accumulated hours was made. They were also asked about ensemble playing. In addition, twin modelling was used to explore the genetic architecture of the relation between musical practice and alexithymia. Alexithymia was negatively associated with (i) musical creative achievement, (ii) having played a musical instrument as compared to never having played, and – for the subsample of participants that had played an instrument – (iii) total hours of musical training (*r* = -0.12 in men and -0.10 in women). Ensemble playing added significant variance. Twin modelling showed that alexithymia had a moderate heritability of 36% and that the association with musical practice could be explained by shared genetic influences. Associations between musical training and alexithymia remained significant when controlling for education, depression, and intelligence. Musical achievement and musical practice are associated with lower levels of alexithymia in both men and women. Musical engagement thus appears to be associated with higher emotional competence, although effect sizes are small. The association between musical training and alexithymia appears to be entirely genetically mediated, suggesting genetic pleiotropy.

## INTRODUCTION

It has been shown repeatedly that musical activity in children and adults is positively associated with performance in a broad range of non-musical cognitive tasks (Schellenberg, 2004, 2006; Schellenberg and Weiss, 2013). There is considerable interest within the cognitive sciences to explore the nature and biological underpinnings of these relations further. Music listening, performance, and composing also involve affective processing (Juslin and Sloboda, 2001), and a similarly important question relates to the potential of associations between musical activity and emotional skills (Wrangsjö and Körlin, 1995; Dissanayake, 2000; Baumgartner et al., 2006; Gabrielsson, 2011).

Emotional competence can be operationalised in different ways, but one key concept in the psychosomatic literature relating emotional ability to disease development is *alexithymia,* – i.e., a deficiency in the ability to process emotions. The alexithymia concept was introduced by Sifneos (Sifneos, 1996) and has been further developed by several authors (Nemiah, 1996). The most frequently used instrument for assessing alexithymia is the Toronto Alexithymia Scale (TAS) consisting of 20 questions TAS-20; Bagby et al., 2014). As expected, alexithymia has been shown to be negatively related to other measures of emotional competence (Baughman et al., 2013).

Several studies have investigated the biological basis of alexithymia. Neuroimaging studies have shown that alexithymia is related to structural (Borsci et al., 2009) and biochemical (14) differences in emotional systems of the brain, as well as to altered patterns of regional brain activity during the processing of emotional stimuli (Duan et al., 2010; Heinzel et al., 2010; Deng et al., 2013), mentalizing (Moriguchi et al., 2006) and imagery (Mantani et al., 2005). Based on a study of 8000 Danish twins Jorgensen et al. (2007) reported a heritability of alexithymia of 30–33% with an additional 12–20% of the variance due to shared environmental influences. These estimates have subsequently been confirmed by two other twin studies (Picardi et al., 2011; Baughman et al., 2013). Alexithymia has been linked to specific polymorphisms of the catechol *O*-methyltransferase (Ham et al., 2005) and serotonin transporter genes (Kano et al., 2012), suggesting that genetically based individual differences in monoamine systems of importance for emotional processing could be one factor underlying alexithymia.

Furthermore, alexithymia has been related to several health outcomes, such as hypertension (Jorgensen and Houston, 1986; Grabe et al., 2010), depression, and reduced likelihood of recovery from alcoholism and substance abuse (Picardi et al., 2011; Evren et al., 2012; de Haan et al., 2013). Further, it has been shown that subjects with early stage hypertension have a relatively poor ability to describe feelings (Theorell et al., 1984). In summary, the literature suggests that alexithymia is a clinically relevant construct. It is associated with differences in brain systems that are used for emotional processing, self-awareness and theory of mind. Individual differences in alexithymia appear to largely depend on genetic factors and some additional shared environment.

The expression and perception of emotions are central elements of music listening and performance (Juslin and Sloboda, 2001). This relates to a more profound and complex question about the functional role of music in human beings and what role emotions may have in this (Madison, 2011). Some researchers have speculated that music may facilitate the experience of emotions and thus be of importance for the development of the ability to identify and differentiate emotions (Dissanayake, 2000; Laiho, 2004; Baumgartner et al., 2006; Gabrielsson, 2011). It therefore appears reasonable that musical engagement may be associated with emotional competence, i.e., lower alexithymia. However, no study to date has explored the relationship between musical activity and alexithymia. Such associations could, if their direction is from music practice to reduced alexithymia, potentially be of interest for intervention approaches. If musical engagement could facilitate emotional development, music education may potentially serve as an intervention strategy to reduce alexithymia, particularly in adolescence (Lichtenstein et al., 2006). However, clearly it may also be the case that alexithymic individuals are simply less likely to seek out musical engagement or that a third factor causes both, alexithymia and low musical engagement.

Here, we use a large sample of adult twins to explore – based on the considerations summarized above – whether alexithymia is negatively associated with active musical engagement operationalized as musical achievement as well as musical practice throughout life time. A reasonable assumption is furthermore that music practice with an ensemble is particularly strongly associated with social interaction. Since emotional and social competence are related to one another, ensemble playing is likely to contribute to the statistical variance in emotional competence. One possibility would be that the two (number of practice hours and experience of ensemble) have additive effects. Another possibility, would be a multiplicative effect, e.g., because emotionally competent individuals are attracted to ensemble playing or because ensemble practice stimulates emotional competence. Therefore we also examined whether playing/singing in an ensemble is associated with lower alexithymia score among those who have ever practiced – with formal tests of multiplicative interaction. Finally, we explored genetic and environmental influences on the relationship between alexithymia and music practice.

## MATERIALS AND METHODS

### PARTICIPANTS

Data were collected as part of a web-survey sent out to a cohort of approximately 32,000 twins born between 1959 and 1985 – the STAGE cohort (Lichtenstein et al., 2002) – from the Swedish Twin Registry (STR), one of the largest registries of its kind (Formann and Piswanger, 1979; Lichtenstein et al., 2002; Magnusson et al., 2013). Zygosity of the twins in the STR has been determined with questions about intra-pair similarities and subsequently was confirmed in 27% of the twins using genotyping, showing that questionnaire based zygosity determination was correct for more than 98% of twin pairs. For further details on the STAGE cohort and zygosity determination in the STR (see Lichtenstein et al., 2002, 2006). The present study received approval from the Regional Ethics Review Board in Stockholm (Dnr 2011/570-31/5, 2011/1425-31, 2012/1107/32). In total, 11,543 individuals participated in the web-survey. For the phenotypic analyses, to control for relatedness within the sample, we randomly selected one twin from each pair and all single twins (*n* = 8599). Of these, 5,881 individuals had valid TAS-20 scores comprising the sample for phenotypic analyses. For the twin analyses we included all individuals with a valid TAS-20 score and zygosity information (*n* = 8, 110), consisting of 1,755 complete twin pairs (851 monozygotic (MZ) and 845 dizygotic (DZ)) as well as 4,600 single twins without the co-twin participating. All participants were aged between 27 and 54 (mean 40.7, SD 7.7).

### MEASURES

#### Music practice

Participants were first asked to indicate whether they had ever played an instrument (including singing). Those who responded positively were questioned about their starting year of practicing and the typical weekly intensity of practicing during four age-intervals (age 0–5, 6–11, 12–17, and 18 till now). From these measures the lifetime total hours of playing was estimated. As expected, music training was positively skewed and kurtosed with many individuals having none or little training. Of the 5,788 participants who had played or sung, 5,777 participants (2,060 men and 3,717 women) reported hours of music practice. Among those who had ever played or sung, the skewness for number of music practice hours was less pronounced than in the total group (skewness 0.80 for men and 1.01 for women). In preliminary analyses, the data were log-transformed and univariate analyses were conducted on both transformed and untransformed data. However, given the large sample size and the fact that the results were very similar with and without log-transformation, untransformed data were used for the final analyses. The approximated total number of training hours in life up to the examination ranged from 0 to 18,400 in men and from 0 to 20,800 in women. More men (*n* = 1,327) than women (*n* = 995) reported that they had never played an instrument.

#### Ensemble playing

“Ensemble playing” was self-assessed by the participant in the larger questionnaire on musical background. Ensemble playing was treated as a dichotomous variable, i.e., participants who reported that they had practiced ensemble playing during any age period were assigned to the “ensemble” group and other subjects to the “no ensemble” group. Alexithymia levels were compared between the playing group and non-playing group.

#### Musical achievement

Musical achievement was assessed used the Music subscale of a Swedish adaptation of the Creative Achievement Questionnaire of Carson (Simonsson-Sarnecki et al., 2000). Musical creative achievement is self-rated using a 7-graded scale, ranging from zero (never) played or sung to seven (nationally or internationally awarded professional).

#### Alexithymia

A back-translated and psychometrically tested Swedish version of TAS-20 (Magnusson Hanson et al., 2014) was used, containing three subscales: the inability to handle emotions due to emotions being poorly recognised (difficulty recognizing), the inability to describe feelings (difficulty describing), and mismatch between coping behaviour and emotions (externally oriented thinking). Here, only the full scale score was used. The scores were normally distributed.

#### Depressive symptoms

Depressive symptoms were measured by means of a six item subscale of the Hopkins symptom checklist (SCL) depression scale. The items are graded from 0–4 giving a range of full scores (sum scores) of 0–24. It has been used in Swedish and Danish population studies (Magnusson Hanson et al., 2014). This was included in the analyses because we wanted to examine whether any putative associations with alexithymia would hold when controlling for depressive symptoms.

#### Education

Level of education was assessed by means of a 10-graded scale reflecting the level of formal school education according to Statistics Sweden. The lowest level was unfinished elementary compulsory school and the highest level an academic doctoral degree. The four lower levels corresponded to no more than high school education whereas the six upper levels corresponded to at least some exposure to college or university education.

#### Wiener Matrizen-Test (WMT)

To estimate general fluid intelligence the Wiener Matrizen-Test (WMT; Carson et al., 2005) was used. The WMT is a timed (maximal test taking time: 25 min.) matrix intelligence test similar to the standard progressive matrices (SPM) from Raven. It consists of 24 multiple choice items. The total score is the number of correctly answered items. The reliability of the WMT has been shown to be relatively high (Cronbach’s alpha = 0.81) and the test correlates well with Raven’s SPM (*r* = 0.92). The WMT score was normally distributed without any outliers.

### STATISTICAL METHODS

#### Group comparisons between practice and non-practice groups

Means of alexithymia scores and estimated total hours of music practice were calculated separately in men and women. Alexithymia scores were compared, using ANCOVA with adjustment for age, in those who reported that they had never played or sung (1,015 men and 708 women) and those who reported that they were playing or singing now or had been doing so in the past (1,512 men and 2,646 women).

#### Analyses within the practice group

In these phenotypic analyses, only men and women who reported that they were playing or singing now or in the past have been included.

(1) *Ensemble playing:* ANCOVA using age adjustment was performed comparing those among the participants who reported having played or sung with experience of ensemble with those in the same group without such experience.
(2) *Number of hours of practice:* In these analyses we explored the effect of hours of practice on alexithymia score.

In order to facilitate interpretation, partial correlations (adjusting for age) analyses were computed not only between alexithymia scores and hours of music practice but also between these main factors and musical achievement, education level, depression scores, and WMT scores. Since 30 correlations were computed for each gender the *p-*value required for significance was set at 0.001 in these correlation analyses.

Since there was a significant correlation between education and alexithymia, separate means of alexithymia scores were also computed for subgroups (with and without college or university education). Similarly significant correlations were found between alexithymia and depressive symptoms. Therefore additional partial correlation analyses (adjusting for each depression and education) were conducted between alexithymia scores and music practice hours. All analyses were performed separately for men and women.

A MANCOVA was performed using ensemble/no ensemble playing and number of musical practice hours divided in tertiles as explanatory variables and alexithymia score as dependent variable with adjustment for age. This allowed us to test whether there was an interaction between ensemble playing and number of practice hours.

#### Genetic analyses based on the full sample

Due to the fact that identical (MZ) twins share all their genes and non-identical (DZ) twins share on average half of their segregating genes, twin studies allow for partitioning of the variance of a trait (and similarly the covariance between two traits) into additive genetic (A), shared environmental (C), and residual (also known as non-shared environmental – E) variation. Variance due to A results from the sum of allelic effects across genes. C results from environmental influences shared by siblings, e.g., home environment, common friends, or socioeconomic status; while E variance results from environmental influences that are not shared between siblings, such as illness or injury and this includes also measurement error. Structural equation modelling Mx (Neale et al., 2006) is employed to estimate the combination of A, C, and E influences that best explains the observed data. For further details on the twin modeling methods or the program used see (Neale and Maes, 2004). Here, a bivariate sex-limitation model was fitted for music practice and alexithymia corrected for age to explore genetic and environmental influences on the covariance between the two traits. The full sample – including subjects who had never practiced – was used for this analysis. The main rationale behind this was statistical power. The number of MZ twins was reduced when the analysis was confined to those who had experience of practice. In addition, since musical achievement and number of hours of music practice were strongly correlated we decided to select one of them – hours of practice – in these analyses.

## RESULTS

### RESULTS OF PHENOTYPIC ANALYSES

Supporting our hypothesis, participants who reported that they had never played an instrument had a significantly higher age adjusted alexithymia mean score (47.5, SD = 9.94, *n* = 1,015 for men and 44.0, SD = 10.5, *n* = 708 for women) than those who reported having ever played (45.8, SD = 9.78, *n* = 1,512 for men and 41.5, SD = 10.2, *n* = 2,646 for women). Further, there were significant differences between the low and high education groups (*p* = 0.001) for both men and women. Also, a higher percentage of men compared to women never engaged in musical practice (40% vs. 21%).

Within the practice group, on average men had higher phenotypic alexithymia scores (*M* = 45.7, SD = 9.8) than women (*M* = 41.5, SD = 10.2).

**Table 1** shows the age-adjusted paired correlations between all study variables separately for men and women in the practice group. The patterns were very similar in men and women therefore in the text we only report the estimates for men (for estimates for both sexes see **Table 1**). *Number of hours of music practice* was significantly and negatively associated with alexithymia (*r* = -0.12) and positively correlated with musical achievement (*r* = 0.52) and education (*r* = 0.10).

**Table 1:** Product moment correlations between the study variables in participants who have ever played or sung (men and women).

(A) Men						
		1	2	3	4	5

1	Music practice hours					
2	Alexithymia score	–0.12*				
3	Musical achievement	0.52*	–0.11*			
4	Education level	–0.10*	–0.17*	–0.11*		
5	Depression score	0.07	0.29*	0.03	–0.01	
6	WMT	0.06	–0.05	0.15*	0.30*	–0.04

*Correlation is significant at the 0.001 level (2-tailed). *n* = 1488 – 1510. Phenotypic correlations (*n* = 1488 – 1510) with random exclusion of one member of each twin pair.						

**(B) Women**						

		1	2	3	4	5

1	Music practice hours					
2	Alexithymia score	–0.10*				
3	Musical achievement	0.41*	–0.10*			
4	Education level	0.08*	–0.18*	0.10*		
5	Depression score	0.04	0.33*	–0.01	–0.02	
6	WMT	0.07*	–0.04	0.14*	0.24*	–0.05

*Correlation is significant at the 0.001 level (2-tailed). Phenotypic correlations (*n* = 2617 – 2639) with random exclusion of one member of each twin pair.

*Alexithymia* was negatively correlated with musical achievement (*r* = -0.11) and education (*r* = -0.17) and positively correlated with depressive symptoms (*r* = 0.29). *Musical achievement* was correlated with education (*r* = 0.11) and with WMT (*r* = 0.15) but unrelated to depressive symptoms. *Depressive symptoms* were unrelated to education and WMT. As expected education was correlated with WMT (*r* = 0.30).

Since there was a significant correlation between education and alexithymia, separate age adjusted marginal means were also computed for the college/no college subgroups (see **Table 2**). Male participants without college or university education reported the highest TAS-20 scores. The remaining three groups had means falling in the following order: men with college education, women without college education, and women with college education. The differences between those without and with college education were highly significant both among men and women (*p* < 0.0001) according to ANCOVA analysis with age correction (with educational group as explanatory and alexithymia score as dependent variable). In addition, the TAS differences between men and women were highly significant in both educational groups (*p* < 0.0001).

**Table 2:** Alexithymia marginal means (adjusted for age) in subgroups (no college/college and no ensemble playing/ensemble playing in men and women.

		Men	Women
No college		47.9 +/-9.9, *n* = 548	44.2 +/-10.4, *n* = 778
College		44.5 +/-9.5, *n* = 963	40.4 +/-9.9, *n* = 1861
			
No ensemble		46.5 +/-9.8, *n* = 1054	42.1 +/-10.3, *n* = 1783
Ensemble		44.1 +/-9.5, *n* = 457	40.1 +/-9.7, *n* = 856

*Differences between no college/college and no ensemble/ensemble separately for men and women are all significant (ANCOVA *p* = 0.0001).*

In line with our second hypothesis, we found significantly lower alexithymia scores among those who played ensemble compared to the other musically active participants, both among men and among women. These differences were highly significant for both men and women (*p* < 0.0001). In addition, the TAS differences between men and women were highly significant both in the ensemble and the no ensemble group (*p* < 0.0001).

**FIGURE 1:**
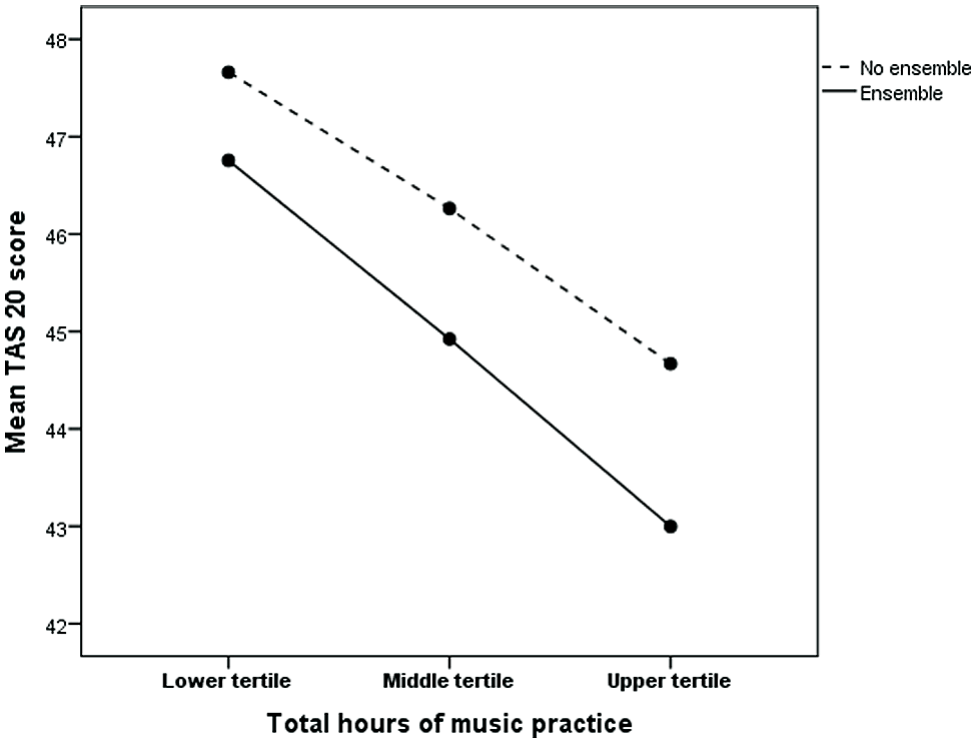
**Age adjusted marginal Toronto Alexithymia Scale (TAS-20) means for ensemble (yes/no) and music practice tertiles among subjects (men) who have practiced playing an instrument.** Music practice tertiles: lower, range 40–800, median 360, Middle, range 840–3160, median 1600, Upper, range 3200–18400, median 5600.

**FIGURE 2:**
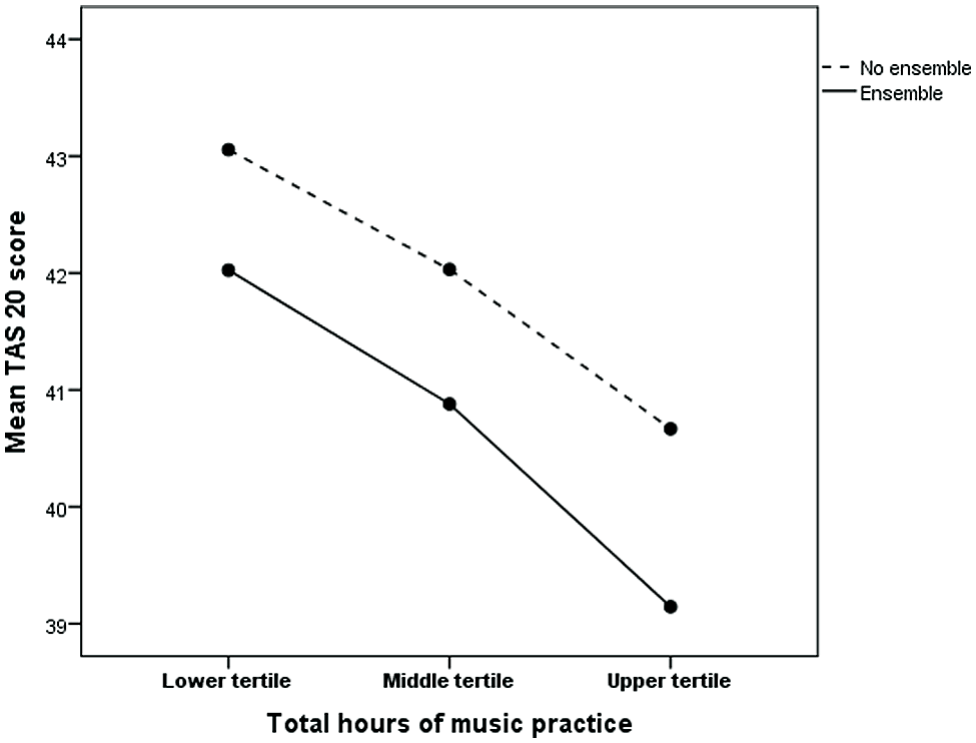
**Age adjusted marginal TAS-20 means for ensemble (yes/no) and music practice tertiles among subjects (women) who have practiced playing an instrument.** Music practice tertiles: lower, range 40–800, median 440, Middle, range 840–2360, median 1360, Upper, range 2400–20800, median 5040.

A MANCOVA using ensemble/no ensemble playing and number of musical practice hours divided in tertiles as explanatory variables, and alexithymia score as dependent variable with age as covariate, showed that both ensemble playing and number of practice hours contributed statistically significantly to a reduced alexithymia score (for men number of hours *p* < 0.0001 and ensemble *p* = 0.032 and for women number of hours *p* < 0.0001 and ensemble *p* = 0.007). There was no interaction between number of hours and ensemble experience in relation to alexithymia. This finding is illustrated in **Figure 1** (men) and **Figure 2** (women). The figure shows that for men the average difference in marginal means between men who have a low number of practice hours (lowest tertile) and no experience in ensemble playing and those who have a high number of practice hours (highest tertile) and experience of ensemble playing is 4.9, corresponding to approximately half a standard deviation. The corresponding difference for women is 4.0.

In a corresponding MANCOVA with age correction it was observed that musical achievement and number of hours of practice contribute statistically independently to alexithymia score (for men number of hours *p* = 0.004 and musical achievement *p* = 0.033, and for women number of hours *p* < 0.0001 and musical achievement *p* = 0.002).

Both education and depressive symptoms were associated with alexithymia. Therefore we tested the effect of controlling for these variables on the correlations between number of music practice hours and alexithymia. The effects were very small (adjustment for education and age *r* = -0.10 in men and *r* = -0.08 in women and with adjustment for depressive symptoms and age *r* = -0.15 for men and *r* = -0.12). WMT did not show a significant association with alexithymia (**Table 1**) and adjusting for WMT score and age left the correlations between number of music practice hours and alexithymia unchanged (*r* = -0.12 for men and *r* = -0.09 for women). Since these results indicated that adjustment for education, depressive symptoms, and WMT score was of minor importance, the genetic analyses were performed without these adjustments.

### RESULTS OF TWIN ANALYSES

**FIGURE 3:**
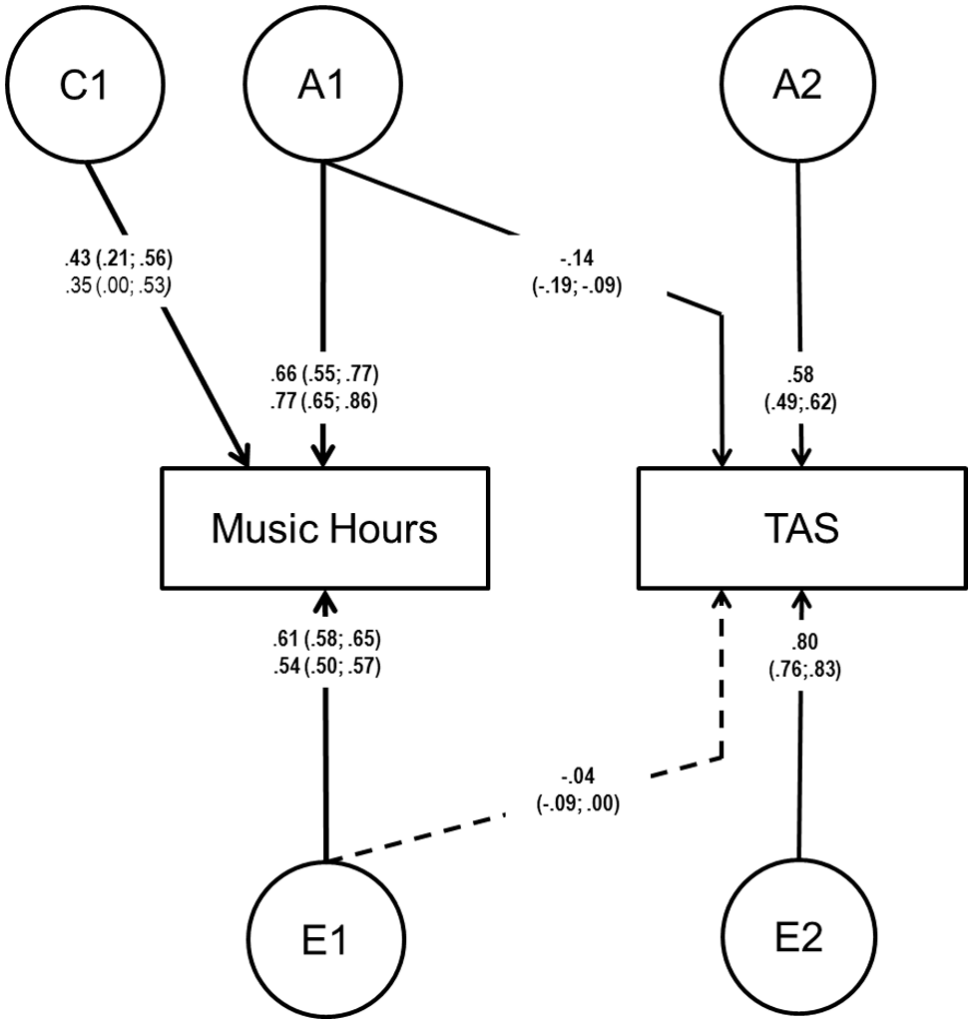
**Reduced sex-limitation model with separate pathways for males (bottom) and females (top) for music practice and TAS with the specific C factor on TAS and the C cross-path dropped.** A = additive genetic; C = common/shared environmental; E = non-shared environmental.

Age and sex adjusted phenotypic correlation between hours of music practice and alexithymia was the same as the one reported above at r = -0.12. **Table 3** shows the twin correlations for hours of music practice and alexithymia. Since the twin correlations were slightly different for men and women, a sex-limitation model was fitted allowing the ACE-estimates to differ between the sexes. Multivariate model fitting results indicated that ACE influences on the TAS as well as the ACE-cross paths between musical practice and TAS could be equated between males and females without a significant deterioration of model fit. Further, all C-influences on the TAS as well as the E-cross path between the two variables could be removed without significant deterioration of the model fit, indicating that an AE model fits the TAS best with all the covariance between the traits being due to shared genetic influences (A). The best fitting model is shown in **Figure 3**. Heritability for the TAS was 36% (27–41%) without significant sex-differences.

**Table 3:** Twin correlations for monozygotic (MZ) and dizygotic (DZ) pairs (female, male, and opposite sex pairs separately) zygosity (bottom) for Toronto Alexithymia Scale and hours of practice corrected for age.

Zygosity	TAS-20	Hours of practice
MZ	0.37 (0.31; 0.42)	0.63 (0.60; 0.66)
DZ	0.13 (0.07; 0.20)	0.40 (0.36; 0.44)
MZ female	0.42 (0.35; 0.49)	0.59 (0.55; 0.63)
MZ male	0.25 (0.14; 0.35)	0.69 (0.65; 0.73)
DZ female	0.12 (0.01; 0.23)	0.44 (0.36; 0.51)
DZ male	0.11 (-0.04; 0.25)	0.44 (0.34; 0.52)
DZOS	0.15 (0.05; 0.25)	0.36 (0.29; 0.42)

*MZ = Monozygotic; DZ = Dizygotic; DZOS = DZ opposite-sex; TAS-20 = Toronto Alexithymia scale.*

## DISCUSSION

The results of the present study suggest that there is a small but significant association between alexithymia and creative achievement in music as well as musical practice. This relationship remained significant even when we controlled for depressive symptoms, education, and cognitive ability, factors that have been shown to be related to participation in activities like music practice (Ahlberg et al., 2004; Picardi et al., 2011). Further we found that ensemble playing was associated with lower alexithymia score compared to other musical engagement which is consistent with past research (Juslin and Sloboda, 2001). Since ensemble playing requires social interaction which requires emotional competence this association could be due either to alexithymia individuals avoiding ensembles and vice versa or to an effect of ensemble playing on the development of emotional competence.

Further, in line with results from a previous similar Danish twin cohort study (Jorgensen et al., 2007; Baughman et al., 2013) we confirmed that about one third of the variance in TAS-20 scores is genetically determined. Our results differ, however, from those in the Danish study as we did not find any significant shared environment effect.

The relationship between musical practice and alexithymia could be explained by shared genes. This indicates that most of the association is due to genetic pleiotropy – the same genes making an individual more susceptible to develop alexithymia also predispose this individual to perform less musical practice and/or vice versa. It is important to note that our findings do not allow for any conclusions regarding causality. However, given that the relationship is due mainly to genetic pleiotropy it is rather unlikely that environmental manipulation of one of the traits would necessarily result in changes in the other trait. It is important to emphasize, however, that a rigorous examination of a causal role of musical practice in preventing alexithymia may require longitudinal controlled studies. The present study is an examination of the association of “volunteer musical practice” – which may be strongly influenced by family selection including genetic factors.

Music is known to induce emotions and it has been proposed that musical activities may contribute to the prevention of alexithymia (Juslin and Sloboda, 2001). There are many examples of music deliberately constructed for the amplification of specific feelings in specific circumstances, for instance music for funerals and solemn or joyful celebration. Such emotional amplification could facilitate development of emotional skills – an emotion is partially “translated” by the music. In such a situation the social context has a decisive role in the emotional effect of the music. Visual impressions may amplify the neurobiological effect of the music and vice versa (Baumgartner et al., 2006). Konecni (2008) have challenged the idea that specific music experiences per se are related to specific emotions. They maintain that “being moved” and “esthetic awe” are the only established psychological reactions to music and that more specific emotional reactions are induced by a combination of musical experience with circumstances and previous experiences. Västfjäll (2001) have described methodology which facilitates the study of the complex interplay between music and emotional responses. In summary it is fair to say that musical experiences may contribute in a complex web of circumstances to specific or more general emotional states.

Ensemble playing is another example of a social musical context. In this study, ensemble practice added to the statistical variance in alexithymia. In future studies one could therefore focus more on the association between ensemble playing and alexithymia. In addition it would be valuable to examine the relationship between other cultural activities, in particular theatre, and alexithymia. A recent Australian quasi-experimental study with control groups showed beneficial effects of increased cultural activities in the school curriculum on emotional and social competence in the pupils (Vaughan et al., 2011). In the same vein, the effects of other kinds of structured emotion teaching should be examined.

### STRENGTHS AND LIMITATIONS

The use of the self-administered TAS-20 builds upon the subjects’ ability to adequately respond to the questions regarding ability to identify emotional states, to give words to them and to act accordingly. Such emotional insight (and ability to fill in the questionnaire adequately) may be gained mainly from people whom the subject interacts with. One may be told by others that one lacks emotional insight. Regardless of which ability the subject has in his/her way of describing this, capacity to deal with emotions may in itself arise in social interaction. It is therefore possible that a self-administered questionnaire of the type that TAS-20 may not capture all aspects of alexithymia. This could lead both to underestimation and overestimation of the true association.

As during recruitment it was clearly indicated that the present study was exploring musical engagement we expect to have a slight over-representation of music-interested individuals in the sample. Apart from that we are confident that our sample represents the Swedish population in the age group 27–54.

## CONCLUSION

This study of a large Swedish twin cohort has shown that musical practice and ensemble playing are significantly albeit weakly associated with reduced alexithymia. The association between musical practice and alexithymia holds even after adjustment for age, education, depressive symptoms, and intelligence and is of the same magnitude in men and women. Accordingly to our twin modelling results, the association between musical practice and alexithymia is genetically determined.

## Conflict of Interest Statement

The authors declare that the research was conducted in the absence of any commercial or financial relationships that could be construed as a potential conflict of interest.
